# Dietary soy and meat proteins induce distinct physiological and gene expression changes in rats

**DOI:** 10.1038/srep20036

**Published:** 2016-02-09

**Authors:** Shangxin Song, Guido J. Hooiveld, Mengjie Li, Fan Zhao, Wei Zhang, Xinglian Xu, Michael Muller, Chunbao Li, Guanghong Zhou

**Affiliations:** 1 Key Laboratory of Meat Processing and Quality Control, MOE; Key Laboratory of Animal Products Processing, MOA; Jiang Synergetic Innovation Center of Meat Processing and Quality Control; Nanjing Agricultural University, Nanjing 210095, P.R. China; 2 Nutrition, Metabolism and Genomics Group, Division of Human Nutrition, Wageningen University, Wageningen, the Netherlands; 3 Norwich Medical School, University of East Anglia Norwich, Norwich, UK; 4 Key Laboratory of Human Function Genomics Jiangsu Province, Nanjing Medical University, Nanjing, 210029, P.R. China

## Abstract

This study reports on a comprehensive comparison of the effects of soy and meat proteins given at the recommended level on physiological markers of metabolic syndrome and the hepatic transcriptome. Male rats were fed semi-synthetic diets for 1 wk that differed only regarding protein source, with casein serving as reference. Body weight gain and adipose tissue mass were significantly reduced by soy but not meat proteins. The insulin resistance index was improved by soy, and to a lesser extent by meat proteins. Liver triacylglycerol contents were reduced by both protein sources, which coincided with increased plasma triacylglycerol concentrations. Both soy and meat proteins changed plasma amino acid patterns. The expression of 1571 and 1369 genes were altered by soy and meat proteins respectively. Functional classification revealed that lipid, energy and amino acid metabolic pathways, as well as insulin signaling pathways were regulated differently by soy and meat proteins. Several transcriptional regulators, including NFE2L2, ATF4, Srebf1 and Rictor were identified as potential key upstream regulators. These results suggest that soy and meat proteins induce distinct physiological and gene expression responses in rats and provide novel evidence and suggestions for the health effects of different protein sources in human diets.

Metabolic syndrome is becoming a global epidemic. It is a cluster of metabolic abnormalities characterized by central obesity, dyslipoproteinemia, hypertension, and glucose intolerance, leading to increased risks of cardiovascular diseases and type 2 diabetes^1^. Dietary intervention has been regarded as an important therapy for metabolic syndrome^2^. For example, dietary restriction^3^ and incorporation of bioactive nutrients (fiber^4^, ω-3 polyunsaturated fatty acids^5^) have been widely studied. Protein is an essential nutrient for the whole life cycle of the human. It has become increasingly recognized that, apart for providing a source of amino acids (AA) for protein synthesis, dietary protein also exerts a wide range of biological functions, such as the regulation of food intake^6^, lipid metabolism^7^, glucose homeostasis and insulin secretion^8^. An example is the soy plant protein which has been recognized as having a hypocholesterolemic effect^9^,^10^. Meat protein is an important dietary source of animal protein for human nutritional requirements. Compared to plant protein, meat protein distinguishes itself for its richness in all the essential amino acids^11^. However, except for their differences in amino acid composition, our understanding regarding the effects of different protein sources on modulating metabolic health is still limited. Therefore, this study was designed to investigate and compare the impacts of different dietary protein sources (soy and meat) in rats on markers of metabolic syndrome (body weight, adipose weight, triacylglycerol (TAG), cholesterol, glucose and insulin levels and the insulin resistance index HOMA-IR). To provide clues for the possible molecular mechanisms that may underlay the effects of the different protein sources, liver gene expression profiles were studied by using RNA-sequence methodology.

## Results

### Growth performance and body composition

**Figure 1:**
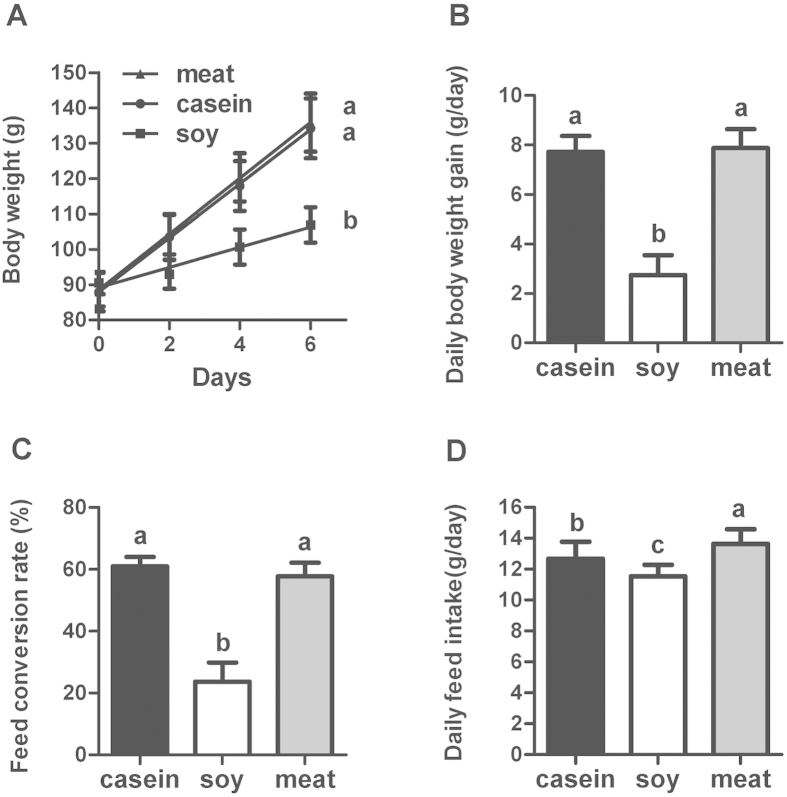
Growth performance of rats fed casein, soy and meat protein diets. (**A**) Body weight; (**B**) Daily body weight gain; (**C**) Feed conversion rate (BWG/FI). (**D**) Daily feed intake. Values are shown as means ± SD. The numbers of biological repetitions of casein, soy and meat protein groups were 10, 10 and 40, respectively. For panel (**A**), linear regression was performed for body weight of rats. The intercept representing initial body weight of rats was not different for three groups. The slope represents growth rate of rats, and its statistical significance (*P* < 0.05) is illustrated by different letters on the right side of lines. For panels (**B**–**D**), different letters above bars indicate significant difference at *P* < 0.05 analyzed by one-way ANOVA and LSD multiple comparisons.

**Figure 2:**
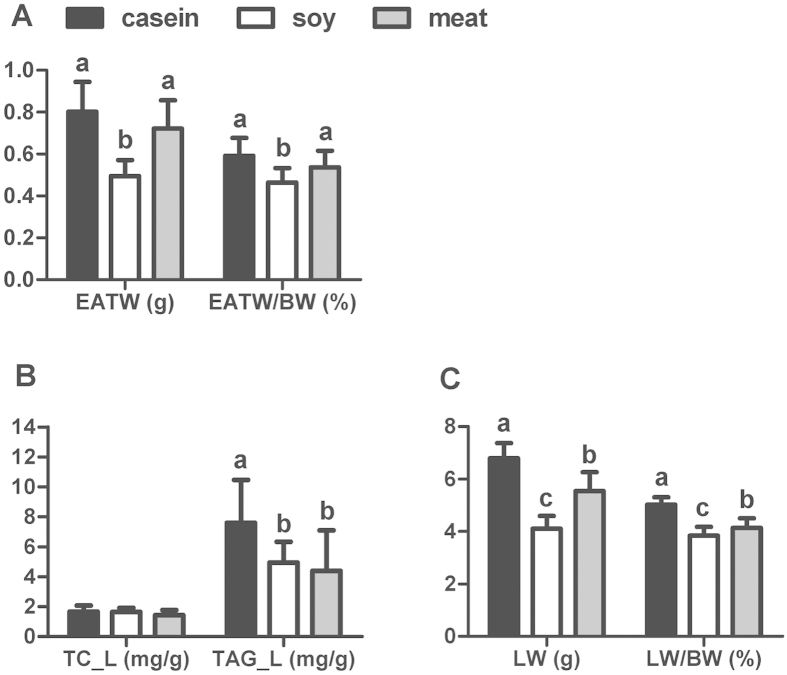
Adipose tissue weight, liver weight, liver TC and TAG content of rats fed casein, soy and meat protein diets. (**A**) EATW: absolute weight of epididymal adipose tissue; EATW/BW: relative weight of epididymal adipose tissue to body weight. (**B**) TC-L: total cholesterol in the liver; TAG-L: triacylglycerol in the liver. (**C**) LW: absolute weight of liver; LW/BW: relative weight of liver to body weight. Values are shown as means ± SD. The numbers of biological repetitions of casein, soybean and meat protein groups were 10, 10 and 40, respectively. Different letters above bars indicate significant difference at *P* < 0.05 tested by one-way ANOVA and LSD multiple comparisons.

Rats in each group had similar initial body weights (Fig. 1). Compared to the casein group, that served as control in our experiment, the group fed dietary soy protein had significantly reduced daily feed intake (decreased by 9%, *P* < 0.05), body weight gain (decreased by 65%, *P* < 0.05) and feed conversion rate (decreased by 61%, *P* < 0.05). However, feeding rats meat protein only increased the daily feed intake (increased by 7.6%, *P* < 0.05), but did not affect the body weight gain or the feed conversion rate (*P* > 0.05). To evaluate the effect of dietary soy and meat proteins on body adiposity, epididymal adipose tissue weight and hepatic lipid content were measured (Fig. 2). Only soy protein reduced the epididymal adipose tissue weight (*P* < 0.05) compared with casein and meat protein. Both soy and meat proteins significantly reduced the liver weight and TAG content (*P* < 0.05). The total cholesterol (TC) content of liver did not differ between the groups.

### Plasma profiling

Plasma TAG concentrations were increased by both soy and meat proteins (*P* < 0.05, Table 1), but only meat protein reduced plasma TC concentrations (*P* < 0.05). Both soy and meat proteins reduced plasma total protein concentrations (*P* < 0.05), but only soy protein increased plasma urea concentrations (increased by 53.7%, *P* < 0.05). Plasma glucose concentrations and insulin levels were significantly reduced by both soy and meat proteins (*P* < 0.05). As a result, the insulin resistance index (HOMA-IR) was improved by both soy and meat proteins (*P* < 0.05). Total plasma AA concentrations were significantly increased only by meat protein (*P* < 0.05, Table 2), and compared to casein this was due to increased concentrations of both nutritionally essential AAs (EAAs) and nonessential AAs (NEAAs) (*P* < 0.05). The only exception was that methionine was reduced by meat protein (reduced by 17.1%, *P* < 0.05). Similarly, feeding soy protein resulted higher plasma concentrations of NEAAs and arginine (*P* < 0.05) compared to casein. However, compared to the meat proteins, the soy protein had much lower concentrations of methionine (decreased by 53.3%, *P* < 0.05) and also valine concentrations were lower (decreased by 21.2%, *P* = 0.054).

### Differentially expressed genes in the liver

**Figure 3:**
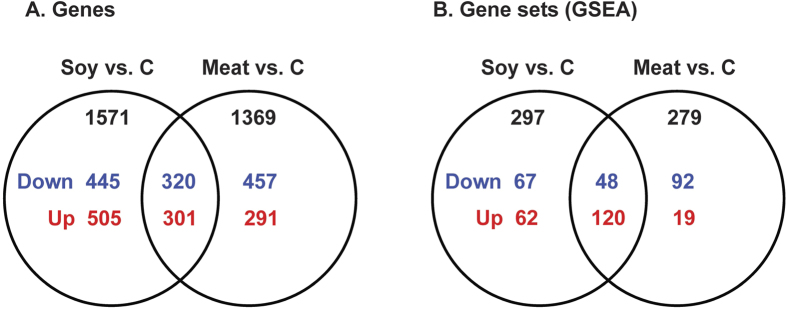
Venn plot of differentially expressed genes and gene sets in the liver of rats fed soy and meat proteins when compared to casein. (**A**) Genes: significant level was set at *P* < 0.05; (**B**) Gene sets (GSEA): significant level was *P* < 0.05 & FDR < 0.25. Soy vs. C: Soy vs. Casein; Meat vs. C: Meat vs. Casein; down: down-regulated; up: up-regulated.

RNA-sequencing was performed to identify differentially expressed genes in rat liver. Soy and meat protein groups were pairwise compared to casein, and the expression of 1571 and 1369 genes were significantly changed by soy and meat proteins, respectively (*P* < 0.05, Fig. 3A). When presented in a Venn plot, it became clear that about half of these changed genes were specific for each protein group. In total, 320 down-regulated genes and 301 up-regulated genes were commonly altered by soy and meat proteins. See Supplementary Table S1 online for all genes.

### Functional implications of differential gene expression

**Figure 4:**
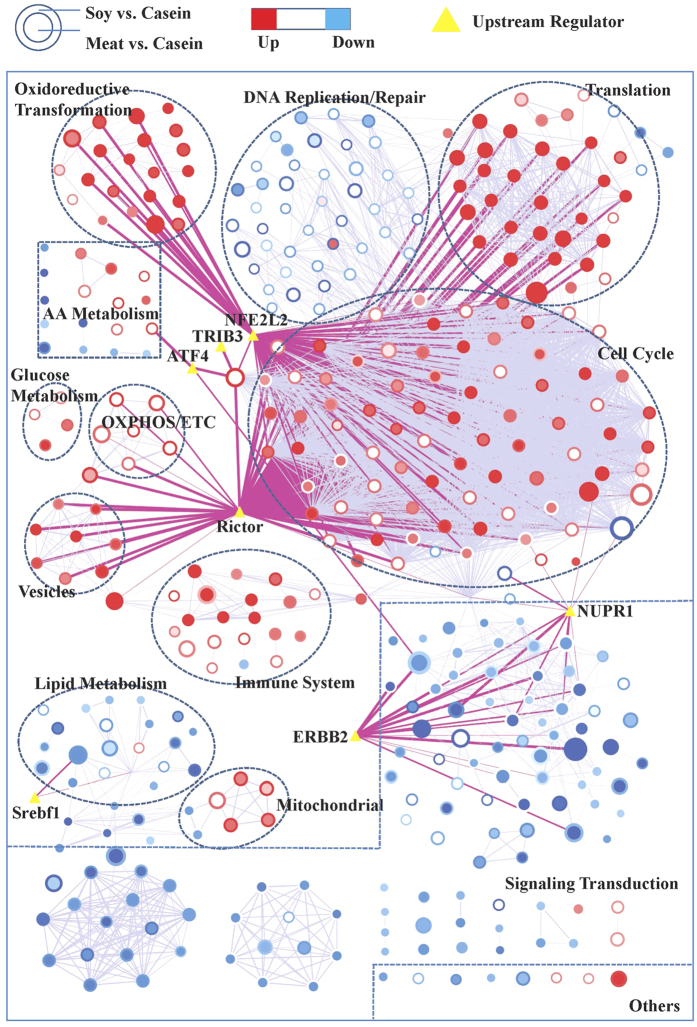
Gene set network of liver transcriptome of the rats fed soy and meat proteins compared to the rats fed casein. The network was produced by using Cytoscape v3.2 and Enrichment Map plugin v2.1. Nodes represent enriched gene sets in GSEA analysis of liver transcriptome. The node size is proportional to the total number of genes within each gene-set (from 15 to 500). The border area of the round node represents the comparison of soy protein group to the casein group (Soy vs. Casein), whereas the inner area of the round node represents the comparison of meat protein group to the casein group (Meat vs. Casein). The colors of nodes indicate the directions of changes of gene-sets with red for up-regulation and blue for down-regulation. The enrichment *P* value is mapped to the node color as a color gradient. The color changes from light to bright with the *P* value decreasing from 0.05 to 0. The weighted grey lines between the round nodes represent the “overlap” score (Jaccard and overlap coefficients >0.375) depending on the number of genes two gene-sets share. The more genes two gene-sets share, the wider the line. The yellow triangles in the map represent the upstream regulators predicted by using Ingenuity Pathway Analysis, and they were overlapped with putative target genes in the genes sets using the Post Analysis tool in the Enrichment Map v2.1 plugin. Pink lines represent the overlap *P* value < 0.05 (Fisher’s Exact Test) between gene sets and upstream regulators. Nodes of high similarity were automatically arranged close together, and circles were semi-automatically annotated and manually labelled.

To gain better insight into the underlying biologic phenomena these changes in gene expression brought about, GSEA was conducted. Compared to the casein group, 297 and 279 gene sets were significantly changed by the dietary soy and meat proteins, respectively, when compared with casein (*P* < 0.05 & FDR < 0.25, Fig. 3B). See Supplementary Table S2 online for all significant gene sets. To enhance the interpretation, all significant gene sets were summarized in an enrichment map, and functionally related gene sets were semi-automatically annotated and manually labeled to highlight the prevalent biologic functions among the related gene sets (Fig. 4). A high-resolution map that includes names of all gene-sets is shown in Supplementary Fig. S1 online. Five clusters of gene sets describing (control of) the cell cycle, mRNA translation, oxidoreductive transformation, immune system and vesicles were increased by both soy and meat proteins compared with the casein diet. Other processes relating to oxidative phosphorylation/electron transport chain and gluconeogenesis were increased by soy protein only. Gene sets relating to AA metabolic pathways were increased by soy protein but were decreased by meat protein. More specifically, gene sets describing the metabolism of methionine, cysteine, phenylalanine, threonine, glycine, serine, alanine, aspartate and glutamine were increased by soy protein only, while gene sets describing the metabolism of tryptophan, histidine and branched-chain amino acids (BCAAs) were repressed by meat protein only.

Gene sets describing lipid metabolism were repressed by both soy and meat proteins (Fig. 4), but to different extents. More specifically, the gene set describing regulation of cholesterol biosynthesis by Srebf (sterol regulatory element-binding transcription factor) was reduced by both soy and meat proteins. Only soy protein reduced expression of genes involved in TAG and cholesterol biosynthesis. Gene sets relating to bile acid biosynthesis were increased by soy protein but reduced by meat protein. In addition, gene sets describing fatty acid degradation (beta oxidation) and biosynthesis were reduced by both soy and meat proteins. However, the PPARα signaling pathway was reduced by meat protein only, which was also confirmed by real-time PCR array analysis. This showed that mRNA expression of 68 PPARα target genes were significantly reduced by meat protein (*P* value < 0.05 & fold change >2.0) but not by soy protein (see Supplementary Table S3 online). In addition, various clusters of signal transduction pathways were suppressed; the TGFβ and focal adhesion pathways were reduced by both soy and meat proteins. Notably, the NOTCH, ERBB, mTOR and insulin signaling pathways were reduced by meat protein only.

### Upstream regulators

To explain the shifts in gene expression profiles, upstream regulator analysis was performed. Results of this analysis identified several transcriptional regulators that were predicted to be activated or inhibited (Table 3). The transcription factor NFE2L2 (nuclear factor erythroid 2-like 2) stood out because it was predicted to be the top activated upstream regulator for both soy and meat proteins. In line with results obtained by GSEA, its target genes overlapped with the cluster of gene sets describing oxidoreductive transformation reactions, but also with some genes involved in (control of) cell cycle. In addition, Rictor (rapamycin-insensitive companion of TOR, complex 2) was predicted to be the top inhibited upstream regulator for both soy and meat proteins and its target genes overlapped with various gene sets relating to cell cycle, translation, as well as oxidative phosphorylation and the electron transport chain. Transcription factor Srebf1 was predicted to be inhibited by soy, and to a lesser extent by meat proteins, and its target genes overlapped with lipid metabolism. Several other regulators were predicted to play a role in the transcriptional responses caused by only one specific dietary protein. Notably, two regulators, activating transcription factor 4 (ATF4) and tribbles pseudokinase 3 (TRIB3), were predicted as being specific for soy protein, and their target genes overlapped with AA metabolism, which was increased by soy protein. On the other hand, PPARG and RXRA were predicted to be upstream regulators for meat proteins only.

## Discussion

We are the first to report on a comprehensive comparison of the effects of dietary soy and meat proteins given at the recommended level on markers of the metabolic syndrome and hepatic mRNA expression. Our work relates to some previously published papers that also reported on the effects of dietary proteins, but most of these studies only focused on lipid metabolism and applied high fat diets in their studies^12^,^13^,^14^,^15^, or focused on only one kind of dietary protein, mostly soy protein^15^ or fish protein^13^,^14^. In addition, other studies investigated the effects of different nutritional levels of dietary protein, such as dietary protein restriction^16^ or a high protein diet^17^, on metabolism in animals. However, unlike these studies, by measuring and comparing the widespread responses on plasma, hepatic gene expression and physiological markers, our study provides a comprehensive comparison between two different protein sources (soy and meat) versus casein, all provided at the recommended level. There are thus several key points which makes our study unique when compared to other studies: 1) In our study the rats were fed nutritionally balanced, semi-synthetic diets (AIN-93G^18^) that differed only regarding protein source; the casein (reference) was fully replaced by purified proteins from soy or meat. 2) We compared the effects of the different dietary protein sources on the physiological and metabolic responses in rats in a comprehensive way, instead of only focusing on specific metabolic or physiological processes such as lipid metabolism. 3) In order to find potential molecular mechanism that may mediate the differential effects, liver gene expression profiles were determined by using RNA-sequence methodology.

Compared to casein, soy and meat proteins induced distinct physiological and molecular effects in rats that suggested an improvement of metabolic health, which is likely caused by the different composition of soy and meat proteins. Soy protein is a mixture of β-conglycinin and glycinin that account for 90% of the total protein^19^. Meat proteins are much more structurally diverse being composed of myofibrillar (50–55%), sarcoplasmic (30–34%) and connective tissue proteins (10–15%)^20^. Compared with casein, these structurally different proteins in soy and meat proteins are likely to digested differently resulting in a diverse range of peptides being absorbed from the gastro-intestinal tract. Our previous study indeed showed that even dietary meat proteins from different animal types were specifically digested, resulting in the production of different peptides in the stomach and jejunum of rats^21^. Except for protein composition, the AA compositions, and particularly the fraction of EAAs in dietary proteins, are the most important criteria and markers for protein quality assessment^22^.

According to the AA compositions found in the protein powders (see Supplementary Table S4 online), diets (see Supplementary Table S5 online) and plasma of rats (Table 2), methionine was found to be the first limiting AA in soy protein. In order to evaluate the AA composition of protein powders, the AA ratios of the EAAs were calculated by using casein +1.5% cysteine (AIN-93G) as the reference protein (see Supplementary Table S6 online)^23^. We found that the AA ratio of methionine in the soy protein was the lowest (52%) among all the EAAs, but in the meat protein it was 111%. Considering the AA composition in the various diets (see Supplementary Table S5 online), methionine was also the lowest EAA (53.7% lower than casein) in the soy protein diet compared to casein group. However, in meat protein diets, the content of methionine was similar to that of the casein group. It has been demonstrated that the plasma AA pattern, in particular the most limiting AA, would cause a direct response when rats are fed an AA imbalanced diet^24^. Further analysis of plasma AA concentrations showed that methionine was significantly reduced by 53.3% by the dietary soy protein, which coincided with the lower methionine content in the soy protein powder and diet. The plasma methionine concentration in the meat protein group was also reduced, compared with casein, but to a lesser extent (reduced by 17.1%). Therefore, we conclude that soy protein is an imbalanced protein source with methionine being the most limiting AA, which is not the case for the meat protein. This is also in accordance with previous observations^11^,^25^.

According to our results, dietary soy protein triggered significant phenotype changes in the rats, including the significantly reduced feed intake and body weight gain. This indicates soy protein may inhibit the appetite and growth of rats, which did not happen to the rats fed meat protein. Correlation analysis found a highly positive correlation between the body weight gain and feed intake (correlation coefficient = 0.8, *P* < 0.05), which indicates the reduced body weight probably resulted from the reduced feed intake induced by the soy protein. At the same time, the soy protein was found to be methionine deficient. It is known that EAA deficiency can lead to feed intake repression, and its mechanism has been thoroughly reviewed^26^. Therefore, we reasoned that the growth inhibitory effect of soy protein is due to the repressed feed intake which may be induced by methionine deficiency. Based on the above, it seems that diet restriction and methionine restriction occurred concomitantly in the soy protein group. However, the rats fed soy protein weighed 20% less than the casein group but consumed only 9% less feed. As a result, the feed intake per gram of body mass (0.65 g/g body weight) was actually higher for the soy protein group than for the casein group (0.57 g/g body weight). Therefore, there was actually no diet restriction on a body weight basis but only the methionine restriction in the soy protein group.

In response to the different protein sources, protein and AA metabolism was regulated differently. Only dietary soy protein increased the metabolism of NEAAs and sulfur-containing AAs in the liver and plasma urea level, indicating increased AA degradation in rats^27^. We believe that this can be related to the methionine limitation in soy protein. In this condition the NEAAs are abundantly available, but since these cannot all be used for body protein synthesis, due to the lack of methionine, they are deaminated to produce urea^24^,^28^. These changes were predicted to be regulated by upstream regulators ATF4 (Z score = 3.498) and TRIB3 (Z score = −2.954). ATF4 is usually up-regulated when AA deprivation occurs^29^ and it regulates a wide array of genes involved in AA transport, metabolism and energy management^30^. TRIB3 acts as a negative feedback regulator of ATF4 and is also involved in the regulation of gene expression in instances of AA limitation^31^. In contrast to soy protein, dietary meat proteins did not change the plasma urea concentrations. Expression level of genes involved in EAA metabolism in the liver were significantly reduced by meat protein only. However, these genes did not overlap with the ATF4 and TRIB3 target genes, indicating that additional molecular mechanisms for AA metabolism are initiated in response to dietary soy and meat proteins.

Our results indicated that both the dietary soy and meat proteins have beneficial effects on lipid metabolism in rats. For the rats fed soy protein, although their feed intakes per gram of body mass were higher than the casein group, their body fat mass including adipose mass and liver TAG content was significantly reduced, which we believe can be related to the significantly repressed lipid and fatty acid biosynthesis in the liver. Unlike soy protein, the dietary meat protein did not reduce adipose mass in rats. As observed for soy protein, the dietary meat protein also reduced the hepatic TAG content and expression of genes involved in lipid synthesis. The transcription factor Srebf1, which is a master regulator of lipogenesis^32^, was predicted to be an inhibited upstream regulator for soy, and to a lesser extent meat proteins. However, expression of genes of the PPARα signaling pathway was only reduced by dietary meat protein according to both the RNA-sequencing and qPCR results. With regards to cholesterol metabolism, dietary soy protein significantly inhibited cholesterol biosynthesis in the liver indicating its potential cholesterol-reducing effect, which has also been reported in previous studies^33^,^34^. However, no significant changes were found in plasma TC levels in rats fed soy protein. This could be because the intervention period in the present study was relatively short compared to previous studies which usually had more than 2-weeks of dietary intervention^33^,^34^. Dietary meat proteins reduced plasma TC levels in rats, indicating a hypocholesterolemic effect of meat protein. Taken together, dietary soy and meat protein may have the similar effects on lipogenesis process via the transcription factor Srebf1 but have different effects on the PPARα signaling pathway in the liver. Both soy and meat protein have beneficial effects on cholesterol homeostasis.

It has been acknowledged that a reduction of body fat occurs when the energy requirements for growth and maintenance exceeds energy intake^35^. Our transcriptomic results revealed that the energy metabolism (oxidative phosphorylation and electron transport chain) in the liver was not affected by dietary meat proteins, and this coincided with the unchanged adipose mass in rats fed meat protein. However, unlike the meat protein group, the hepatic expression of genes involved in oxidative phosphorylation and electron transport chain were significantly increased in the rats fed soy protein. At the same time, these genes significantly overlapped with the target genes of Rictor, which was predicted to be the top inhibited upstream regulator for the soy protein (Z score = −6.090). Rictor is a key regulatory/structural subunit of mTORC2 and plays an important role in regulating hepatic glycolysis and lipogenesis via glucokinase and Srebf1^36^. Therefore, we speculate that the body fat reducing effect of the soy protein may be related to the increased energy metabolism and decreased lipogenesis mediated through the transcription regulators Rictor and Srebf1.

Insulin resistance is a major component of metabolic syndrome^37^. A previous study suggested that soy protein may reduce insulin secretion by the pancreas which in turn was related to the plasma amino acid pattern after consumption of soy protein^38^. However, some epidemiological studies have suggested that animal protein may increase risk of diabetes^39^. Uhe *et al.* (1992) observed that ingestion of beef, chicken or fish proteins did not change insulin level in human subjects^40^. In the present study we found that plasma insulin levels were significantly reduced by both soy and meat proteins compared to the casein group, indicating that both soy and meat proteins have potential effects on improving insulin sensitivity.

Taken together, our results showed that dietary soy and meat proteins induce widespread but distinct physiological and gene expression responses in rats. These responses may be attributed to the different AA contents in soy and meat protein sources, especially of the EAA. Soy protein was shown to be deficient in methionine. According to various methionine restriction studies, the biological responses to methionine restriction are variable^41^,^42^,^43^. Despite this heterogeneity, many of the biological processes that were changed by the dietary soy protein in our study were similar to the results reported in previous methionine restriction studies^41^,^42^,^43^. These commonly changed biological processes included the increased feed intake per gram of body weight, the reduced body weight gain, adiposity and lipid metabolism, and the increased energy metabolism and insulin sensitivity^41^,^42^,^43^. At the same time, our transcriptomic results indicated that the mTOR (mammalian target of rapamycin) signaling pathway was significantly changed by the dietary meat protein (Supplementary Fig. S1 online). It has been recognized that mTOR is a master sensor of amino acid levels in cells and regulates widespread biological processes including protein synthesis and autophagy^44^. Furthermore, other research suggests that many pathways, such as mTOR, insulin, PPAR and Srebf signaling, have close crosstalk in regulating the cellular responses to cope with changes in nutrient status or stress^45^,^46^. Based on the above, these widespread physiological and gene expression responses to different dietary protein sources found in our study are biologically relevant. Our study thus provides important novel scientific evidence and suggestions for the health effects of different protein sources in human diets.

## Materials and Methods

### Ethics statement

Male *Sprague Dawley* (SD) rats were purchased from the Shanghai Laboratory Animal Research Center, Chinese Academy of Science at 3 weeks of age. The animals were housed in pairs in a controlled environment with a 12h light-dark cycle (12:12 h reversed light/dark cycle, 23 °C). The animal experiment was performed according to the guide for care and use of laboratory animals of Nanjing Agriculture University (Nanjing, China) and the Jiangsu Provincial Academy of Agricultural Sciences (The license number was SCXK (Su) 2002–0029). All efforts were made to minimize the number of animals used and to minimize their suffering. The rats were acclimated for 1 week before the study and they had free access to water and standard rat chow throughout the experiment.

### Diets

The standard, nutritionally balanced semi-synthetic AIN-93G^18^ was used as reference diet in this study. The protein source in this diet is casein (milk protein). The other five experimental diets (soy, beef, pork, chicken and fish protein) were also prepared according to the AIN-93G formula, except that casein was fully replaced by one of the five proteins (Table 4). To ensure consistency between the diets, large batches of ingredients of diets were purchased from Dyets Inc. (Bethlehem, PA). Soy protein isolates (food grade) were purchased from Linyi Shansong biological products company (Linyi, China) and treated with 80% methanol to remove isoflavones. Meat protein sources were prepared as follows: raw meat materials, i.e., pork and beef *Longissimus dorsi* muscle, chicken *pectoralis major* muscle and fish (carp) dorsal muscle were cooked in a 72 °C water bath to an internal temperature of 70 °C. The cooked meat were chilled to 4 °C and at 16h, the cooked meat was freeze-dried and twice defatted with methylene chloride/methanol (2:1, v:v). After the fat extraction, the residual solvent was removed by evaporation and the resulting protein powder was passed through a 30 Mesh (0.595 mm) sieve. The final protein powders consisted of more than 90% of protein and 6–9% of water. AA composition of protein powders (see Supplementary Table S4 online) and diets (see Supplementary Table S5 online) was determined using a Hitachi L-8900 AA analyzer (Tokyo, Japan). The presence of iron in meat results from it being a component of the heme in myoglobin and thus, as there are large differences in contents of different meats, iron was not balanced during diet preparation. Other minerals in the diets were balanced based on the actual mineral composition in protein powders (see Supplementary Table S7 online). The mineral formulations for the six diets are listed in the Supplemental Table S8 online. All diets were prepared by Jiangsu-Xietong, Inc. (Nanjing, China).

### Experimental protocol

When the rats arrived at the laboratory, all rats were fed the standard AIN-93G diet for 7 days to adapt to the purified diets and new environment. Subsequently, the rats were randomly allocated to one of the six diet groups (n = 10 per group), i.e. casein, soy, pork, beef, chicken or fish protein groups. Rats were fed these diets for 7 days. Body weights and dietary intakes were measured every 2 days. On the day of sacrifice, rats were deprived of feed for 4 h prior to sacrifice but were given free access to water. Rats were anaesthetized with ether inhalation. Blood was taken by orbital puncture and plasma was isolated. Liver and epididymal adipose tissues were obtained, weighed and snap frozen in liquid nitrogen. All samples were stored at −80 °C until analysis. To simplify the comparison between the soy and meat proteins versus casein, all data from the four meat protein groups, including the physiological, plasma and gene expression data, were combined and analyzed as single meat protein group.

### Liver lipid contents and plasma parameters detection

TAG and TC contents in the liver were determined using commercial kits purchased from Nanjing Jiancheng Bioengineering Institute (Nanjing, China). Plasma TAG, TC, glucose, urea, and total protein concentrations were analyzed using a Hitachi 7180 auto analyzer (Tokyo, Japan). Plasma insulin concentrations were determined using a radioimmunoassay kit purchased from Beijing North Institute of Biological Technology (Beijing, China). The homeostasis model assessment for insulin resistance (HOMA-IR)^47^ was calculated according to the equation IR = (fasting insulin in mU/L × fasting glucose in mM)/22.5. Plasma free AA concentrations were determined using a Hitachi L-8900 AA analyzer (Tokyo, Japan).

### Gene expression profiling in liver by RNA-sequencing

#### RNA isolation, library construction and sequencing

Total liver RNA was isolated by using the RNAiso Reagent Kit (Takara, Dalian, China). RNA-sequencing of three biological replicates from each group was commissioned to BGI Tech (Shenzhen, China). In brief, the isolated RNA samples were treated with DNase I to remove DNA, and enriched by using oligo (dT) magnetic beads. The mRNA was fragmented into short fragments (about 200 bp), and converted to double strand cDNA. The cDNA was purified with magnetic beads, and sequencing adaptors were ligated to the fragments. The fragments were enriched by PCR amplification. The library products were sequenced on an Illumina HiSeq 2000 machine (BGI, Shenzhen, China).

#### Identification of differentially expressed genes

After cleaning up the raw sequence data by removing adapter sequences and low quality reads, reads were quantified and annotated using the workflow implemented in the Bioconductor package QuasR^48^. Reads were mapped against the Rat Genome Sequencing Consortium Rnor_5.0 rat genome assembly, and annotated based on the University of California Santa Cruz known Genes track^49^,^50^. Nonspecific filtering of the count table was carried out to increase detection power^51^, based on the requirement that a gene should have an expression level greater than 1 count per million reads mapped for at least 3 libraries across all samples. Differences in library size were adjusted for by the trimmed mean of M-values normalization method^52^, implemented in the Bioconductor package edgeR^53^. Counts were then log-transformed and the observed mean-variance trend was converted into precision weights by the voom function^54^ in the Bioconductor package *limma*^55^. Differentially expressed genes were identified by using linear models and a moderated t-statistic^55^. Genes that satisfied the criterion of *P* < 0.05 were considered to be significantly regulated.

### Biological interpretation of gene expression data

Changes in gene expression were related to biologically meaningful changes using gene set enrichment analysis (GSEA)^56^. It is well accepted that GSEA has multiple advantages over analyses performed on the level of individual genes^56^,^57^. Gene-sets were retrieved from the expert-curated Kyoto Encyclopedia of Genes and Genomes, Biocarta, Reactome and WikiPathways pathway databases. Only gene-sets consisting of more than 15 and fewer than 500 genes were taken into account, which resulted in the inclusion of 2,272 gene sets. For each comparison, genes were ranked on their t-value that was calculated by the empirical Bayes method. Statistical significance of GSEA results was determined using 1,000 permutations. The Enrichment Map v2.1.0 plugin for Cytoscape v3.2.0 was used for visualization and interpretation of the GSEA results^58^. Enrichment maps were generated with gene-sets that passed conservative significance thresholds (*P* < 0.05, False Discovery Rate (FDR) < 0.25). Upstream regulator analysis in Ingenuity Pathway Analysis (content version 21249400 released 23 September 2014; Ingenuity Systems) was used to identify the cascade of potential upstream regulators that may explain the observed gene expression changes in the data set. For this study, focus was on transcriptional regulators. Target genes of several top regulators were overlapped to the genes in the gene set network using Post Analysis tool in Enrichment Map v2.1.0.

### Quantitative PCR array analyzing PPAR signaling pathway

The PPAR (peroxisome proliferator-activated receptor) signaling pathway was profiled using RT^2^ profiler PCR array (330231 PARN-149ZA, Qiagen, Hilden, Germany), according to the manufacturer’s instructions. The RT^2^ Profiler PCR array profiles the expression of 84 target genes besides housekeeping and control genes. Gene amplification was performed on an Applied Biosystems 7500 Real-Time PCR System (Foster City, CA).

### Statistical methods

Except for the RNA-seq data, all statistical analyses were performed using the SPSS version 16.0 (Chicago, IL). The diet effect on measured variables were analyzed by one-way analysis of variance (ANOVA) and means were compared by least-significant difference (LSD) multiple comparison. Statistical significance was set at *P* < 0.05. Values are shown as means ± SD.

## Additional Information

**How to cite this article**: Song, S. *et al.* Dietary soy and meat proteins induce distinct physiological and gene expression changes in rats. *Sci. Rep.* **6**, 20036; doi: 10.1038/srep20036 (2016).

## Supplementary Material

Supplementary Information

Supplementary Dataset 1

Supplementary Dataset 2

## Figures and Tables

**Table 1:** Plasma triacylglycerol, cholesterol, glucose, insulin, total protein and urea concentrations of rats fed casein, soy or meat protein diets.

	Casein (n = 10)	Soy (n = 7)^1^	Meat(n = 39)^2^
TAG, mmol/L	0.35 ± 0.06^b^	0.45 ± 0.08^a^	0.43 ± 0.06^a^
TC, mmol/L	2.06 ± 0.41^a^	1.99 ± 0.21^ab^	1.84 ± 0.25^b^
glucose, mmol/L	8.51 ± 0.63^a^	6.89 ± 1.69^b^	7.44 ± 1.56^b^
insulin, mIU/L	32.39 ± 23.27^a^	10.77 ± 6.43^b^	18.31 ± 12.46^b^
HOMA-IR	12.14 ± 8.15^a^	3.66 ± 2.71^b^	6.53 ± 5.31^b^
total protein, g/L	51.51 ± 3.86^a^	45.76 ± 3.12^b^	48.65 ± 3.93^b^
urea, mmol/L	3.91 ± 0.66^b^	6.01 ± 1.43^a^	3.88 ± 1.31^b^

Values are shown as means ± SD. (n = 7)^1^, blood of three rats in soy group was collected but yielded insufficient plasma to allow analyses; (n = 39)^2^, blood of one rat in meat group was collected but yielded insufficient plasma to allow analyses. Different superscripts indicate statistical significance at *P* < 0.05 analyzed by one-way ANOVA and LSD multiple test. TAG, triacylglycerol; TC, total cholesterol; HOMA-IR was calculated using the formula: HOMA-IR = [glucose (mmol/L) × insulin (mIU/L)/22.5], using fasting values.

**Table 2:**
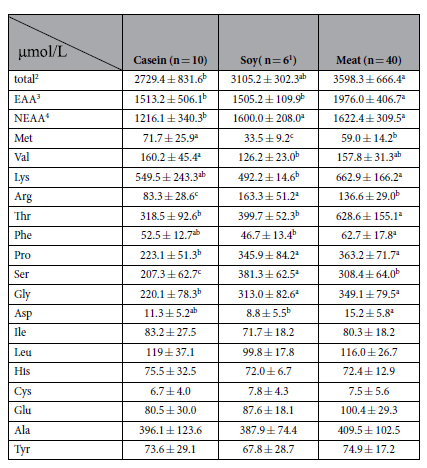
Plasma amino acid concentrations of the rats fed casein, soy or meat protein diets.

Values are shown as means ± SD. (n = 6)^1^, blood of four rats in soy group was collected but yielded insufficient plasma to allow analyses. Different superscripts indicate the statistical significance at *P* < 0.05 analyzed by one-way ANOVA and LSD multiple test. Total^2^: the sum of 17 kinds of amino acids in plasma including Arg, Pro, Met, Val, Ser, Gly, Lys, Thr, Phe, Asp, Ile, Leu, Cys, Glu, Ala, Tyr, His. EAA^3^: the sum of 9 kinds of essential amino acids in plasma including Arg, Met, Val, Lys, Thr, Phe, Ile, Leu, His. NEAA^2^: the sum of 8 kinds of non-essential amino acids in plasma including Pro, Ser, Gly, Asp, Cys, Glu, Ala, Tyr.

**Table 3:** Upstream regulators predicted for the hepatic transcriptome changes in the rats fed soy or meat protein diets compared to rats fed casein.

UpstreamRegulator	Molecule Type	Z Score^1^	
		Soy vs.newline/>Casein	Meat vs.newline/>Casein
NFE2L2	transcription regulator	4.368	3.273
NUPR1	transcription regulator	2.814	2.699
ATF4	transcription regulator	3.498	
SPDEF	transcription regulator	2.646	
EIF4E	translation regulator	2.311	
PTTG1	transcription regulator		2.213
PPARG	ligand-dependent nuclear receptor		2.377
RXRA	ligand-dependent nuclear receptor		−2.458
CTNNB1	transcription regulator	−2.055	
MLXIPL	transcription regulator	−2.407	
CCND1	transcription regulator	−2.414	
FOXM1	transcription regulator	−2.533	
TRIB3	kinase	−2.954	
ERBB2	kinase	−2.310	−3.075
Srebf1	transcription regulator	−2.956	−2.179
Rictor	transcription regulator	−6.090	−4.308

All upstream regulators were significant at *P* < 0.01 as calculated by the Fisher’s Exact Test. Z score^1^: determine whether an upstream regulator is significantly activated (>2.0) or inhibited (<−2.0).

NFE2L2: nuclear factor, erythroid 2-like 2; NUPR1: nuclear protein 1; ATF4: activating transcription factor 4; SPDEF: SAM pointed domain-containing Ets transcription factor; EIF4E: Eukaryotic translation initiation factor 4E; PTTG1: Securin; PPARG: Peroxisome proliferator-activated receptor gamma; RXRA: Retinoid X receptor alpha (RXR-alpha); CTNNB1: Catenin beta-1; MLXIPL: Carbohydrate-responsive element-binding protein/MLX-interacting protein-like; CCND1: Cyclin-D1; FOXM1: forkhead box protein M1; TRIB3: tribbles pseudokinase 3; ERBB2: v-erb-b2 avian erythroblastic leukemia viral oncogene homolog 2;Srebf1: sterol regulatory element-binding transcription factor 1; Rictor: rapamycin-insensitive companion of TOR, complex 2.

**Table 4:** Ingredient composition and nutritional content of diets.

g/Kg diet	Casein	Soy	Pork	Beef	Chicken	Fish
diet composition, g/Kg diet						
Protein^1^	200.0	203.0	190.0	195.0	192.0	191.0
Cornstarch	397.5	397.5	397.5	397.5	397.5	397.5
Dyetros	132	132	132	132	132	132
Sucrose	100	100	100	100	100	100
Soybean oil	70	70	70	70	70	70
Cellulose	50	50	50	50	50	50
Mineral mix^2^	35.0	31.9	30.3	33.4	31.4	29.2
Vitamin mix^3^	10	10	10	10	10	10
L-Cystine^4^	3.0	0	0	0	0	0
Choline Bitartrate	2.5	2.5	2.5	2.5	2.5	2.5
nutritional level, U/Kg						
Energy, Kcal	4056.0	4056.0	4056.0	4056.0	4056.0	4056.0
Protein, g	177	177	177	177	177	177
Fat, g	70	70	70	70	70	70
Carbohydrate, g	679.5	679.5	679.5	679.5	679.5	679.5

Protein^1^, the amount of protein powder was adjusted and balanced according to the protein content in soy and meat protein powder. Mineral mix^2^, the formulation of mineral mixes for the six diets is listed in Supplementary Table S8 online. Vitamin mix^3^: the formulation of vitamin mix as described 18. L-Cystine^4^: the amino acid composition of soy and meat protein diets were not modified.
