# Supplementary material for: Dietary soy and meat proteins induce distinct physiological and gene expression changes in rats
Source: Sci Rep. 2016 Feb 9;6:20036. doi: 10.1038/srep20036 (PMC4746574; doi:10.1038/srep20036)
Supplement: Supplementary Information [file srep20036-s1.pdf]

Online Supplementary Materials

Dietary soy and meat proteins induce distinct physiological and gene expression responses in rats

Shangxin Song[1], Guido J. Hooiveld[2], Mengjie Li[1], Fan Zhao[1], Wei Zhang[4], Michael Muller[3], Chunbao Li[1]*, Guanghong Zhou[1]*

[1]Key Laboratory of Meat Processing and Quality Control, MOE; Key Laboratory of Animal Products Processing, MOA; Synergetic Innovation Center of Food Safety and Nutrition; Jiang Synergetic Innovation Center of Meat Processing and Quality Control; Nanjing Agricultural University; Nanjing 210095, P.R. China

[2]Nutrition, Metabolism and Genomics Group, Division of Human Nutrition, Wageningen University, Wageningen, the Netherlands

[3]Norwich Medical School, University of East Anglia Norwich, Norwich, UK

[4]Key Laboratory of Human Function Genomics Jiangsu Province, Nanjing Medical University, Nanjing, 210029, P.R. China

***Corresponding authors**

Dr. Guanghong Zhou

Address: Weigang 1#, Nanjing, 210095, P.R. China. E-mail: guanghong.zhou@hotmail.com; Tel: 86 25 84395376; Fax: 86 25 84395679

Dr. Chunbao Li

Address: Weigang 1#, Nanjing, 210095, P.R. China. E-mail: chunbao.li@njau.edu.cn; Tel/fax: 86 25 84395679

Online Supplementary Materials

Supplementary Figure S1. Network with all names of gene sets and upstream regulators for liver transcriptome of the rats fed soy and meat proteins compared to the rats fed casein.

This network was produced by using Cytoscape v3.2 and Enrichment Map plugin v2.1. Nodes represent enriched gene sets in gene set enrichment analysis of liver transcriptome in rat. The node size is proportional to the total number of genes within each gene set (from 15 to 500). The border area of the node represents the comparison of soy protein group to the casein group (Soy vs. Casein), whereas the inner area of the node represents the comparison of meat protein group to the casein group (Meat vs. Casein). The colors of nodes indicate the directions of changes of gene sets with red for up-regulation and blue for down-regulation. The enrichment *P* value is mapped to the node color as a color gradient. The color changes from light to bright with the P value decreasing from 0.05 to 0. The weighted grey lines between the round nodes represent the ‘‘overlap’’ score (Jaccard and overlap coefficients>0.375) depending on the number of genes two gene-sets share. The more genes two gene-sets share, the wider the line. The yellow triangles in the map represent the upstream regulators predicted by using Ingenuity Pathway Analysis, and they were overlapped with putative target genes in the genes sets using the Post Analysis tool in the Enrichment Map v2.1 plugin. Pink lines between round nodes and yellow triangles represent the overlap *P* value (Fisher’s Exact Test nominal *P*<0.05) between the enriched gene sets and the upstream regulators. Nodes of high similarity were automatically arranged close together, and circles were semi-automatically annotated and manually labelled.

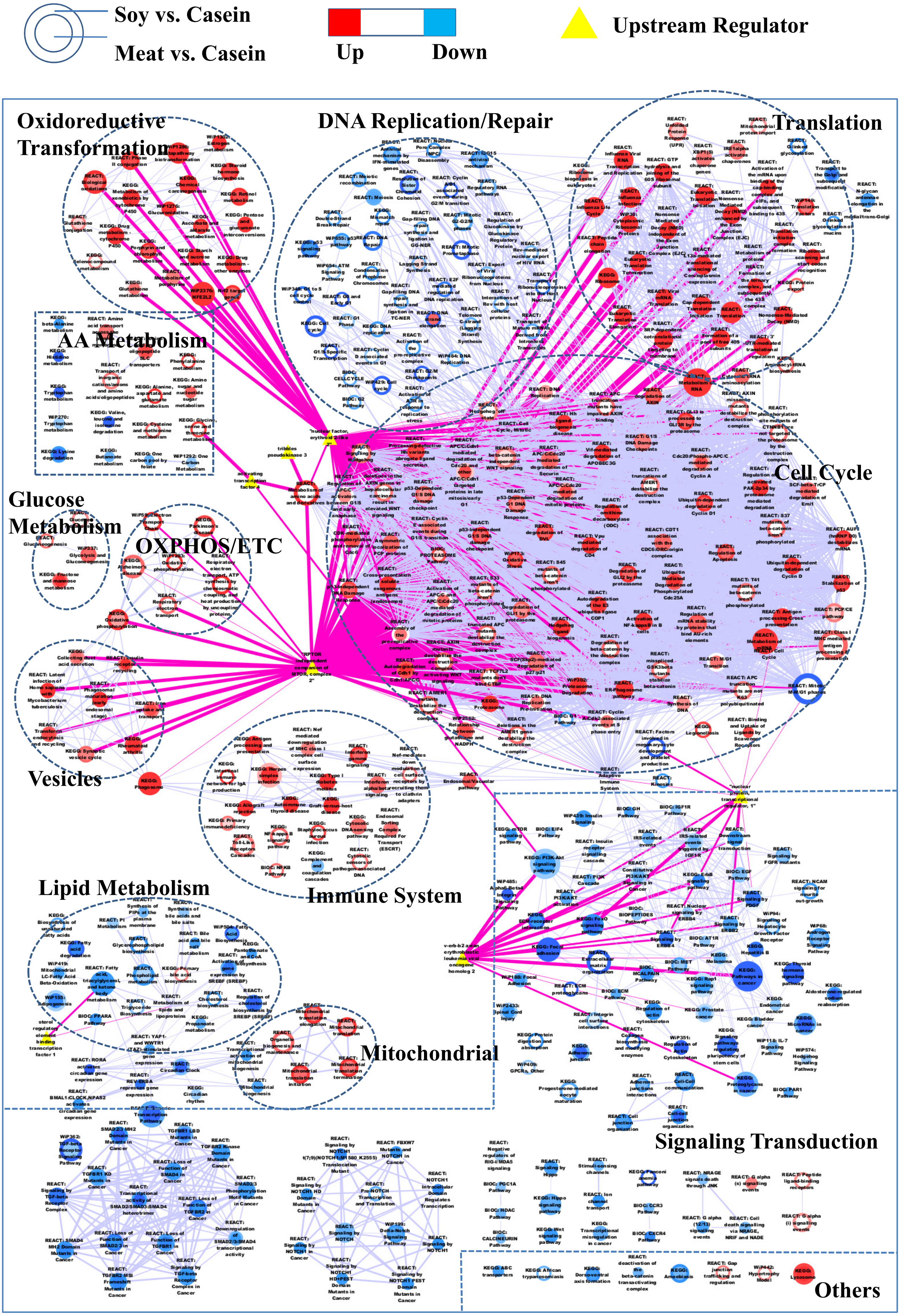
Soy vs. Casein
Meat vs. Casein
Up
Down
Upstream Regulator
Oxidoreductive Transformation
DNA Replication/Repair
Translation
AA Metabolism
Glucose Metabolism
OXPHOS/ETC
Cell Cycle
Vesicles
Immune System
Lipid Metabolism
Mitochondrial
Signaling Transduction
Others

Online Supplementary Materials

Supplementary Table S1. All significantly regulated genes (*P* <0.05) observed in rats after 1 week consumption of the soy and meat protein diets compared to the casein diet.

Due to the length of Supplemental Table S1, it is available as a separate supplemental Excel file.

Supplementary Table S2. All significantly gene sets (*P* <0.05 & FDR<0.25) obtained from the GSEA analysis of the liver transcriptomic data observed in rats after 1 week consumption of the soy and meat protein diets compared to the casein diet.

Due to the length of Supplemental Table S2, it is available as a separate supplemental Excel file.

Online Supplementary Materials

Supplementary Table S3. The mRNA expression of PPAR target genes in rat livers after 1 week consumption of the soy and meat protein diets compared to the casein diet.

		Soy vs. Casein		Meat vs. Casein	
**Symbol**	**Full name**	**FC**	***P***	**FC**	***P***
Scd1	stearoyl-Coenzyme A desaturase 1	-16.80	0.24	-3.56	0.060
Txnip	thioredoxin interacting protein	-13.51	0.35	-11.03	0.038
Aqp7	aquaporin 7	-6.53	0.09	-3.67	0.0042
Fabp5	fatty acid binding protein 5, epidermal	-6.31	0.23	-2.52	0.065
Angptl4	angiopoietin-like 4	-5.48	0.07	-6.00	0.00089
Fabp1	fatty acid binding protein 1, liver	-4.97	0.08	-2.85	0.0053
Ehhadh	enoyl-Coenzyme A, hydratase/3-hydroxyacyl Coenzyme A dehydrogenase	-4.63	0.16	-3.16	0.033
Hprt1	hypoxanthine phosphoribosyltransferase 1	-4.24	0.26	-3.29	0.025
Apoc3	apolipoprotein C-III	-4.21	0.15	-3.68	0.0024
Klf10	kruppel-like factor 10	-4.08	0.13	-2.95	0.012
Acadl	acyl-Coenzyme A dehydrogenase, long-chain	-4.08	0.14	-3.05	0.010
Acsl3	acyl-CoA synthetase long-chain family member 3	-3.90	0.03	-3.32	0.00057
Acsl1	acyl-CoA synthetase long-chain family member 1	-3.82	0.14	-3.37	0.0053
Slc27a5	solute carrier family 27 (fatty acid transporter), member 5	-3.82	0.17	-3.00	0.020
Cyp27a1	cytochrome P450, family 27, subfamily a, polypeptide 1	-3.72	0.22	-3.73	0.0083
Etfdh	electron-transferring-flavoprotein dehydrogenase	-3.70	0.21	-2.90	0.012
Pten	phosphatase and tensin homolog	-3.53	0.29	-2.93	0.013
Cpt2	carnitine palmitoyltransferase 2	-3.44	0.27	-3.36	0.0059
Apoa1	apolipoprotein A-I	-3.43	0.23	-3.01	0.010
Cpt1a	carnitine palmitoyltransferase 1a, liver	-3.42	0.29	-3.42	0.015
Slc22a5	solute carrier family 22 (organic	-3.32	0.27	-2.52	0.066

	cation/carnitine transporter), member 5				
Clu	clusterin	-3.32	0.28	-3.73	0.0036
Ppargc1b	peroxisome proliferator-activated receptor gamma, coactivator 1 beta	-3.23	0.12	-2.85	0.026
Slc27a2	solute carrier family 27 (fatty acid transporter), member 2	-3.17	0.18	-2.71	0.011
Acadm	acyl-Coenzyme A dehydrogenase, C-4 to C-12 straight chain	-3.17	0.17	-3.60	0.0023
Acsl5	acyl-CoA synthetase long-chain family member 5	-3.15	0.19	-2.50	0.039
Ppargc1a	peroxisome proliferator-activated receptor gamma, coactivator 1 alpha	-3.15	0.15	-2.35	0.077
Eln	elastin	-3.15	0.19	-2.54	0.028
Ech1	enoyl coenzyme A hydratase 1, peroxisomal	-3.14	0.31	-2.43	0.035
Sirt1	sirtuin (silent mating type information regulation 2 homolog) 1 (S. cerevisiae)	-3.14	0.22	-3.45	0.0043
Acox1	acyl-Coenzyme A oxidase 1, palmitoyl	-3.12	0.15	-3.39	0.0059
B2m	beta-2 microglobulin	-3.03	0.33	-2.70	0.022
Hmgcs2	3-hydroxy-3-methylglutaryl-Coenzyme A synthase 2 (mitochondrial)	-2.99	0.22	-3.32	0.0073
Ppara	peroxisome proliferator activated receptor alpha	-2.96	0.30	-2.82	0.027
Ep300	e1A binding protein p300	-2.96	0.28	-4.47	0.0052
Fads2	fatty acid desaturase 2	-2.96	0.15	-2.71	0.017
Ppard	peroxisome proliferator-activated receptor delta	-2.95	0.21	-2.95	0.010
Actb	actin, beta	-2.95	0.28	-3.09	0.0045
Ilk	integrin-linked kinase	-2.94	0.31	-3.15	0.012
Fabp2	fatty acid binding protein 2, intestinal	-2.83	0.21	-2.32	0.061
Acaa2	acetyl-Coenzyme A acyltransferase 2	-2.70	0.18	-2.96	0.0053
Mlycd	malonyl-CoA decarboxylase	-2.69	0.35	-3.45	0.016
Hif1a	hypoxia-inducible factor 1, alpha subunit (basic helix-loop-helix transcription factor)	-2.68	0.34	-2.946	0.012
Lpin1	lipin 1	-2.66	0.12	-2.80	0.024

Pdpk1	3-phosphoinositide dependent protein kinase-1	-2.65	0.30	-3.14	0.011
Crebbp	CREB binding protein	-2.63	0.30	-3.15	0.0060
Fabp7	fatty acid binding protein 7, brain	-2.54	0.19	-2.81	0.013
Ldha	lactate dehydrogenase A	-2.51	0.22	-2.69	0.0061
Fgr	Gardner-Rasheed feline sarcoma viral (v-fgr) oncogene homolog	-2.49	0.26	-3.08	0.0086
Nr1h3	nuclear receptor subfamily 1, group H, member 3	-2.47	0.31	-3.00	0.0082
Tgs1	trimethylguanosine synthase homolog (S. cerevisiae)	-2.41	0.20	-2.62	0.020
Rxra	retinoid X receptor alpha	-2.40	0.18	-2.68	0.0064
Hspd1	heat shock protein 1 (chaperonin)	-2.28	0.28	-2.09	0.020
Apoe	apolipoprotein E	-2.23	0.36	-2.89	0.013
Apoa5	apolipoprotein A-V	-2.18	0.53	-3.35	0.0080
Med1	mediator complex subunit 1	-2.15	0.27	-2.65	0.020
Rxrg	retinoid X receptor gamma	-2.13	0.37	-2.68	0.011
Acox3	acyl-Coenzyme A oxidase 3, pristanoyl	-2.13	0.26	-2.87	0.0070
Dgat1	diacylglycerol O-acyltransferase homolog 1 (mouse)	-2.06	0.40	-2.61	0.020
Slc27a1	solute carrier family 27 (fatty acid transporter), member 1	-1.97	0.30	-1.18	0.63
Acsl4	acyl-CoA synthetase long-chain family member 4	-1.93	0.42	-2.61	0.019
Fabp4	fatty acid binding protein 4, adipocyte	-1.87	0.47	-2.87	0.0080
Ncoa6	nuclear receptor coactivator 6	-1.82	0.49	-2.69	0.011
Creb1	CAMP responsive element binding protein 1	-1.78	0.35	-2.38	0.0090
Pck1	phosphoenolpyruvate carboxykinase 1 (soluble)	-1.74	0.53	-2.54	0.019
Pltp	phospholipid transfer protein	-1.71	0.41	-2.32	0.045
Rxrb	retinoid X receptor beta	-1.69	0.42	-2.01	0.069
Ncoa3	nuclear receptor coactivator 3	-1.69	0.45	-2.15	0.036
Cd36	CD36 molecule (thrombospondin receptor)	-1.63	0.65	-1.84	0.26
Gk	glycerol kinase	-1.61	0.65]	-2.85	0.014

Pprc1	peroxisome proliferator-activated receptor gamma, coactivator-related 1	-1.56	0.94	-1.88	0.44
Lpl	lipoprotein lipase	-1.50	0.85	-2.22	0.13
Src	v-src sarcoma (Schmidt-Ruppin A-2) viral oncogene homolog (avian)	-1.48	0.44	-2.08	0.017
Sorbs1	sorbin and SH3 domain containing 1	-1.48	0.35	-1.98	0.0096
Fabp3	fatty acid binding protein 3, muscle and heart	-1.41	0.44	-1.45	0.74
Slc27a4	solute carrier family 27 (fatty acid transporter), member 4	-1.26	0.79	-1.69	0.16
Rplp1	ribosomal protein, large, P1	-1.19	0.93	-1.84	0.067
Smarcd3	SWI/SNF related, matrix associated, actin dependent regulator of chromatin, subfamily d, member 3	-1.14	0.56	-1.29	0.24
Cyp7a1	cytochrome P450, family 7, subfamily a, polypeptide 1	-1.11	0.80	-2.94	0.023
Mmp9	matrix metallopeptidase 9	-1.05	0.36	-1.05	0.042
Olr1	oxidized low density lipoprotein (lectin-like) receptor 1	-1.04	0.36	-1.04	0.042
Pyy	peptide YY (mapped)	-1.04	0.36	-1.04	0.042
Pparg	peroxisome proliferator-activated receptor gamma	1.25	0.40	-1.70	0.16
Cpt1b	carnitine palmitoyltransferase 1b, muscle	1.38	0.42	1.03	0.73
Pck2	phosphoenolpyruvate carboxykinase 2 (mitochondrial)	1.43	0.35	-1.90	0.091
Ucp1	uncoupling protein 1 (mitochondrial, proton carrier)	1.66	0.36	1.16	0.63

FC: fold change; *P*: statistical significance analyzed by T test was set at 0.05.

Online Supplementary Materials

Supplementary Table S4. The amino acid compositions of the protein powders

%	Casein[1]	Soy	Pork	Beef	Chicken	Fish
Met	2.6	1.34±0.06 ^{a}	2.89±0.09 ^{b}	2.78±0.13 ^{b}	2.86±0.23 ^{b}	2.99±0.01 ^{b}
Lys	7.4	5.67±0.21 ^{a}	8.47±0.29 ^{b}	8.75±0.11 ^{bc}	8.67±0.58 ^{bc}	9.10±0.09 ^{c}
Pro	11.7	4.42±0.21 ^{d}	3.21± 0.12 ^{bc}	3.43±0.04 ^{c}	2.90±0.19 ^{a}	2.98±0.03 ^{ab}
Val	5.7	3.06±0.17 ^{a}	3.17±0.19 ^{b}	3.12±0.07 ^{b}	2.90±0.28 ^{b}	3.06±0.06 ^{b}
Thr	3.8	3.43±0.20 ^{a}	4.48±0.20 ^{b}	4.53±0.11 ^{b}	4.30±0.27 ^{b}	4.30±0.03 ^{b}
Arg	3.6	7.01±0.44 ^{b}	6.30±0.36 ^{a}	6.43±0.12 ^{ab}	6.27±0.41 ^{a}	5.94±0.10 ^{a}
Asp	6.5	10.23±0.45 ^{c}	9.08±0.36 ^{ab}	9.11±0.14 ^{ab}	9.00±0.62 ^{a}	9.78±0.03 ^{bc}
Gly	1.8	3.74±0.16 ^{a}	3.96±0.18 ^{ab}	4.15±0.14 ^{bc}	3.76±0.23 ^{a}	4.33±0.05 ^{c}
Ser	5.4	4.60±0.32 ^{b}	3.79±0.23 ^{a}	3.82±0.14 ^{a}	3.71±0.16 ^{a}	3.88±0.09 ^{a}
Phe	5	4.70±0.16 ^{b}	3.89±0.14 ^{a}	3.92±0.04 ^{a}	3.79±0.29 ^{a}	3.99±0.04 ^{a}
Ile	4.8	3.90±0.10 ^{a}	4.33±0.09 ^{ab}	4.39±0.09 ^{b}	4.33±0.48 ^{ab}	4.10±0.10 ^{ab}
Leu	8.8	7.16±0.21 ^{a}	7.98±0.23 ^{b}	8.07±0.07 ^{b}	7.75±0.72 ^{ab}	7.88±0.17 ^{b}
Cys	0.4	1.36±0.11 ^{b}	0.86±0.08 ^{a}	0.92±0.27 ^{a}	0.76±0.11 ^{a}	0.88±0.02 ^{a}
Glu	20.8	18.22±1.34 ^{b}	15.47±1.03 ^{a}	16.12±0.74 ^{a}	14.94±0.58 ^{a}	15.35±0.45 ^{a}
Ala	2.6	3.85±0.17 ^{a}	5.46±0.23 ^{b}	5.64±0.07 ^{b}	5.47±0.34 ^{b}	5.65±0.03 ^{b}
Tyr	5.3	4.14±0.16 ^{a}	4.64±0.16 ^{a}	4.58±0.07 ^{a}	4.60±0.30 ^{a}	4.58±0.02 ^{a}
His	2.6	2.32±0.09 ^{a}	3.66±0.14 ^{c}	3.00±0.02 ^{b}	2.90±0.20 ^{b}	2.41±0.01 ^{a}

[1] values for casein were obtained from the company Dyets Inc. (Bethlehem, PA). Values are shown as means ± SD. Different superscripts mean statistically significant difference at P<0.05 tested by one-way ANOVA and LSD multiple comparisons.

Online Supplementary Materials

Supplementary Table S5. The amino acid compositions of the diets

%	AIN-93G	Soy	Pork	Beef	Chicken	Fish
Ala	0.62±0.01[a]	0.76±0.01[b]	1.06±0.01[c]	1.13±0.02[d]	1.09±0.02[c]	1.12±0.01[d]
Arg	0.71±0.01[a]	1.31±0.02[d]	1.15±0.02[b]	1.23±0.02[c]	1.2±0.02[c]	1.13±0.00[b]
Asp	1.42±0.02[a]	2.03±0.03[e]	1.77±0.01[b]	1.84±0.03[c]	1.79±0.04[b]	1.95±0.02[d]
Cys	0.37±0.03[c]	0.22±0.01[b]	0.15±0.01[a]	0.14±0.01[a]	0.15±0.00[a]	0.15±0.01[a]
Glu	4.85±0.09[d]	3.81±0.05[c]	3.16±0.07[a]	3.44±0.05[b]	3.15±0.06[a]	3.25±0.03[a]
Gly	0.37±0.01[a]	0.74±0.01[b]	0.78±0.01[c]	0.83±0.01[d]	0.75±0.01[b]	0.87±0.01[e]
His	0.55±0.01[c]	0.45±0.01[a]	0.69±0.01[e]	0.59±0.01[d]	0.56±0.01[c]	0.47±0.00[b]
Ile	0.97±0.02[e]	0.78±0.02[a]	0.85±0.01[bc]	0.89±0.02[d]	0.86±0.02[cd]	0.83±0.01[b]
Leu	1.92±0.03[d]	1.47±0.02[a]	1.6±0.02[b]	1.69±0.02[c]	1.6±0.03[b]	1.63±0.02[b]
Lys	1.57±0.02[b]	1.07±0.02[a]	1.6±0.00[b]	1.69±0.03[c]	1.66±0.03[c]	1.76±0.01[d]
Met	0.54±0.00[c]	0.25±0.01[a]	0.51±0.01[b]	0.53±0.01[c]	0.51±0.01[b]	0.54±0.00[c]
Phe	1.01±0.01[e]	0.95±0.02[d]	0.77±0.01[a]	0.85±0.01[c]	0.77±0.01[a]	0.82±0.01[b]
Pro	2.16±0.01[e]	0.92±0.01[d]	0.67±0.03[b]	0.74±0.00[c]	0.64±0.02[a]	0.64±0.01[a]
Thr	0.87±0.01[b]	0.69±0.01[a]	0.88±0.00[b]	0.92±0.02[c]	0.87±0.02[b]	0.86±0.01[b]
Ser	1.17±0.01[d]	0.94±0.01[c]	0.77±0.00[a]	0.79±0.01[b]	0.77±0.02[a]	0.80±0.01[b]
Tyr	0.80±0.01[c]	0.45±0.01[b]	0.41±0.02[a]	0.44±0.03[ab]	0.41±0.01[a]	0.44±0.00[ab]
Val	1.24±0.03[c]	0.80±0.02[a]	0.89±0.01[b]	0.90±0.02[b]	0.89±0.01[b]	0.90±0.00[b]

Values are shown as means ± SD. Different letters mean statistically significant difference at $P<0.05$ tested by one-way ANOVA and LSD multiple comparisons.

Online Supplementary Materials

Supplementary Table S6. Amino acid ratios[1] of soy and meat proteins using casein+1.5%Cys (AIN93G) as the reference protein

%	Soy	Meat[2]	Pork	Beef	Chicken	Fish
Met	52	111	111	107	110	115
Val	54	54	56	55	51	54
Ile	81	89	90	91	90	85
Leu	81	90	91	92	88	90
BCAA[3]	73	79	80	81	78	78
Lys	77	118	114	118	117	123
His	89	115	141	115	112	93
Thr	90	116	118	119	113	113
Phe	94	78	78	78	76	80
Arg	195	173	175	179	174	165

Amino acid ratios[1] (%) = (mg of an essential amino acid in 1.0 g of test protein/mg of the same amino acid in 1.0 g of (casein + 1.5% Cys)) ×100; Meat[2]: it was calculated by combining value of meat proteins from pork, beef, chicken and fish together. BCAA[3]: it was calculated by combining value of Val, Ile and Leu together.

Online Supplementary Materials

Supplementary Table S7. Mineral composition of the protein powders

mg/Kg	Casein[1]	Soy	Pork	Beef	Chicken	Fish
Ca	500.0	707.3±15.4 ^{c}	209.4±101.1 ^{a}	176.8±56.5 ^{a}	158.6±5.9 ^{a}	440.4±104.0 ^{b}
Cu	4.0	13.2±0.2 ^{c}	4.9±1.3 ^{b}	2.9±1.3 ^{a}	2.1±0.5 ^{a}	1.8±0.9 ^{a}
Cr	0.8	0.6±0.0	nd[2]	0.9±0.4	0.8±0.1	0.7±0.1
Fe	7.0	150.4±7.6 ^{c}	35.7±1.9 ^{a}	78.0±25.6 ^{b}	20.3±5.5 ^{a}	18.0±2.2 ^{a}
K	50.0	389.3±7.9 ^{a}	9944.0±125.4 ^{d}	4438.8±183.1 ^{b}	6411.2±60.2 ^{c}	13375.5±267.6 ^{e}
Mg	30.0	405.6±3.7 ^{a}	983.2±30.3 ^{c}	676.8±32.6 ^{b}	1163.0±22.2 ^{d}	1187.8±35.6 ^{d}
Mn	1.6	11.4±0.1	0.7±0.3	nd	0.6±0.1	0.8±0.1
Na	100.0	8184.9±142.8 ^{d}	1192.4±202.4 ^{c}	486.0±108.2 ^{a}	685.0±101.1 ^{ab}	848.2±56.8 ^{b}
Ni	0.2	1.6±0.4	0.8±0.3	nd	0.8±0.2	nd
P	7500.0	6788.4±93.4 ^{b}	7217.4±351.7 ^{cd}	4741.4±147.8 ^{a}	7547.6±114.5 ^{d}	7131.5±201.0 ^{bc}
Se	0.5	nd	7.4±2.2	3.9±0.7	8.2±0.8	7.8±0.9
Zn	38.0	43.9±14.0 ^{a}	86.8±14.2 ^{b}	161.5±11.6 ^{c}	44.4±2.5 ^{a}	27.6±3.3 ^{a}

Minerals in protein powders were detected using inductively coupled plasma optical emission spectrometer (PerkinElmer, Optima 2100 DV). Casein[1]: Value for casein was obtained from company Dyets Inc. (Bethlehem, PA); nd[2]: not detected. Values for soy and meat proteins are shown as means ± SD. Different superscripts mean statistically significant difference at $P<0.05$ tested by one-way ANOVA and LSD multiple comparisons.

Online Supplementary Materials

Supplementary Table S8. Ingredient composition of mineral mixes of the six diets

Ingredients, g/Kg	Casein	Soy	Pork	Beef	Chicken	Fish
calcium carbonate, anhydrous, Ca 40.04%	357.00	390.17	320.43	326.06	371.41	268.99
potassium dihydrogen phosphate, P 22.76% K 28.73%	196.00	208.48	113.59	209.56	182.48	38.50
calcium phosphate, Ca 23.29% P 18.00%	0.00	0.00	166.18	90.08	54.27	274.10
potassium Citrate, K 36.16%	70.78	78.37	0.00	0.00	0.00	0.00
sodium chloride, Na 39.34%	74.00	0.00	68.16	71.80	73.42	76.24
potassium sulphate, S 18.39% K 44.87%	46.60	51.61	53.83	48.79	51.91	55.78
magnesium oxide, Mg 60.32%	24.00	22.02	17.85	18.90	15.29	16.22
iron citrate, Fe 16.5%	6.06	0.00	0.00	0.00	0.00	0.00
zinc carbonate, Zn 52.14%	1.65	1.67	1.34	0.35	1.77	2.12
manganous carbonate, Mn 47.79%	0.63	0.54	0.74	0.68	0.71	0.76
curpic carbonate, Cu 57.47%	0.30	0.21	0.34	0.32	0.35	0.38
potassium iodate, I 59.3%	0.01	0.01	0.01	0.01	0.01	0.01
sodium selenate, Se 41.79%	0.01025	0.01025	0.00000	0.00000	0.00000	0.00000
ammonium paramolybdate, 4 hydrate, Mo 54.34%	0.00795	0.00880	0.00918	0.00832	0.00886	0.00952
sodium meta-silicate, 9 hydrate, Si 9.88%	1.45	1.61	1.67	1.52	1.62	1.74
chromium potassium sulfate, 12 hydrate, Cr 10.42%	0.275	0.305	0.318	0.288	0.306	0.329
lithium chloride, Li 16.38%	0.0174	0.0193	0.0201	0.0182	0.0194	0.0208
boric acid, B 17.5%	0.0815	0.0903	0.0941	0.0853	0.0908	0.0976
sodium fluoride, F 45.24%	0.0635	0.0703	0.0733	0.0665	0.0707	0.0760
nickel carbonate, Ni 45%	0.0318	0.0352	0.0367	0.0333	0.0354	0.0381
ammonium vanadate, V 43.55%	0.0066	0.0073	0.0076	0.0069	0.0074	0.0079
suger	221.026	244.772	255.311	231.420	246.216	264.566
total	1000.00	1000.00	1000.00	1000.00	1000.00	1000.00
